# Colorectal Cancer Classification and Cell Heterogeneity: A Systems Oncology Approach

**DOI:** 10.3390/ijms160613610

**Published:** 2015-06-15

**Authors:** Moisés Blanco-Calvo, Ángel Concha, Angélica Figueroa, Federico Garrido, Manuel Valladares-Ayerbes

**Affiliations:** 1 Translational Cancer Research Group, Instituto de Investigación Biomédica de A Coruña (INIBIC), Complexo Hospitalario Universitario de A Coruña (CHUAC), Servizo Galego de Saúde (SERGAS), Universidade da Coruña (UDC), As Xubias, 84, 15006 A Coruña, Spain; E-Mails: Moises.Blanco.Calvo@sergas.es (M.B.-C.); Angel.Concha.Lopez@sergas.es (A.C.); Angelica.Figueroa.Conde-Valvis@sergas.es (A.F.); 2 Pathology Department, Complexo Hospitalario Universitario A Coruña (CHUAC), Servizo Galego de Saúde (SERGAS), As Xubias, 84, 15006 A Coruña, Spain; 3 Departamento de Bioquímica, Biología Molecular III e Inmunología, Facultad de Medicina, Universidad de Granada, Avenida de Madrid, s/n, 18012 Granada, Spain; E-Mail: federico.garrido.sspa@juntadeandalucia.es; 4 UGC de Labortorio Clínico, Hospital Universitario Virgen de las Nieves, Servicio de Análisis Clínicos, Avenida de las Fuerzas Armadas, 2, 18009 Granada, Spain; 5 Instituto de Investigacion Biosanitaria de Granada (IBISGranada), Avenida de las Fuerzas Armadas, 2, 18009 Granada, Spain;; 6 Medical Oncology Department, Complexo Hospitalario Universitario A Coruña (CHUAC), Servizo Galego de Saúde (SERGAS), As Xubias, 84, 15006 A Coruña, Spain

**Keywords:** colorectal cancer, classification, heterogeneity, cancer systems biology, molecular pathology, targeted therapy, precision medicine

## Abstract

Colorectal cancer is a heterogeneous disease that manifests through diverse clinical scenarios. During many years, our knowledge about the variability of colorectal tumors was limited to the histopathological analysis from which generic classifications associated with different clinical expectations are derived. However, currently we are beginning to understand that under the intense pathological and clinical variability of these tumors there underlies strong genetic and biological heterogeneity. Thus, with the increasing available information of inter-tumor and intra-tumor heterogeneity, the classical pathological approach is being displaced in favor of novel molecular classifications. In the present article, we summarize the most relevant proposals of molecular classifications obtained from the analysis of colorectal tumors using powerful high throughput techniques and devices. We also discuss the role that cancer systems biology may play in the integration and interpretation of the high amount of data generated and the challenges to be addressed in the future development of precision oncology. In addition, we review the current state of implementation of these novel tools in the pathological laboratory and in clinical practice.

## 1. Introduction

**Figure 1:**
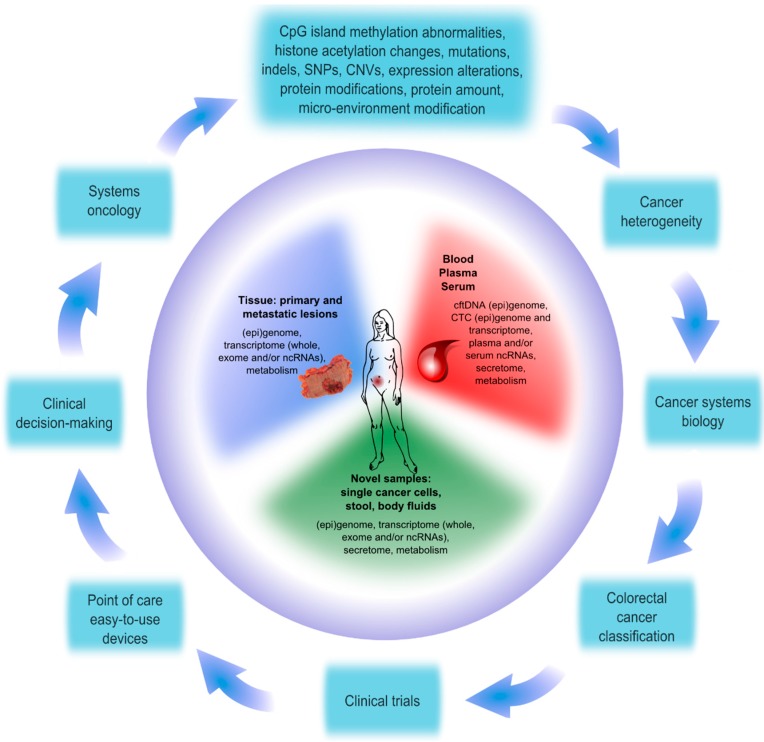
The study of colorectal cancer can be addressed using many different sample sources beyond biopsies from primary tumors. Additional sample sources include metastatic tissue, blood, and derivatives (plasma and serum), as well as other body fluids, stool or single cancer cells. Using high throughput technologies, within these samples we can analyze tumor DNA, circulating free DNA (cftDNA), circulating tumor cells (CTCs), RNA and proteins in order to discover alterations in (epi) genome, transcriptome, secretome, metabolism, and so on. These alterations include CpG island methylation, histone modification, mutations, insertion and deletion events (indels), single nucleotide polymorphisms (SNPs), copy number variations (CNVs), changes in the amount of proteins and RNAs, and so on. All these alterations contribute together to tumor heterogeneity, and to improve the overall understanding of colorectal cancer, cancer systems biology should integrate and link them to each other. Once integrated, we will be able to develop molecular classifications to better define the possible outcome scenarios and the therapeutic strategies to follow with each patient individually. Each classification should be tested in properly designed clinical trials, and in the future, if the classification demonstrates usefulness, point-of-care devices could be developed in order to apply the novel tools to facilitate clinical decision-making. This would be a definite step towards the implementation of systems oncology, which should be continually improved with more analyses in more samples.

Colorectal cancer is a complex disease with a variable clinical course and with important divergences in the response to treatment, even in tumors with similar histopathological characteristics. Today we know that the most plausible explanation for this erratic behavior may reside in the strong heterogeneity both between and inside of tumors. Within tumors, not only do many different families or clones of cancer cells coexist [1] but also each cancer cell shows important dissimilarities regarding remaining cells due to the presence of different genetic and biological alterations [2]. In addition, tumors are considered as highly dynamic entities, subjected to intense evolutionary pressure, in which cell composition, biological phenotype, and clinical characteristics are continually evolving over time [3]. Therefore, in order to enhance the understanding of tumor biology, we need novel methods to obtain samples in multiple spatial and temporal points in the course of the disease. We need also novel strategies to study all these samples at higher resolutions, dissecting the molecular nature of each cancer cell through the analysis of numerous parameters at multiple levels (genes, mRNA, non-coding RNAs, proteins, metabolism, and so on). Finally, we should develop tools to allow the integration of a huge amount of data so we can obtain a holistic, systemic view of the abnormalities behind the malignant behavior of tumors. Nowadays, with the advances in analytical capacities, we have already started to better understand the causes and consequences of tumor heterogeneity [4]. As a result, the classical pathological approach for colorectal cancer classification is being gradually overcome in favor of more precise and multi-parametric molecular classifications [5]. The information so obtained could be used in the future to provide the proper treatment for each patient and to develop tools to monitor the evolution and response of the disease (Figure 1).

## 2. Sources of Heterogeneity in Colorectal Cancer

During the development of adenocarcinomas, a number of molecular, cellular, and histological alterations give rise to the transition from normal epithelium to adenoma and ultimately to cancer. These alterations can be caused both by genetic and non-genetic events that, in turn, can be classified into deterministic and stochastic. While the deterministic events (such as variations in the transcriptional activity of different genes) contribute to the generation of cell subtypes with phenotype and physiology similar to those found in normal tissues, the stochastic events (such as transcriptional noise or variations in the amount of signaling components) account for the cell-to-cell heterogeneity [2]. Even when these alterations may account for most of the heterogeneity within tumors, they have been poorly studied compared to genetic events, which have been extensively analyzed. In the case of colorectal cancer, genetic alterations are basically attributed to genomic instability [4], which can operate through three general ways [6]: chromosomal instability (CIN), microsatellite instability (MSI) and CpG island methylator phenotype (CIMP). During the development of colorectal cancer, several of these alterations can appear separately or in combination (e.g., CIMP usually appears with MSI, due to the methylation and silencing of mismatch DNA repair genes), generating thus a first level of inter-tumor heterogeneity [7]. In fact, the different combinations of these molecular alterations lead to the development of tumors with different clinical and pathological characteristics so that patients can be classified by function of their prognosis and management [8,9,10].

Beyond this first approach to the understanding of colorectal cancer biology, the detailed knowledge on the specific abnormalities within tumors remained elusive until the recent developments of high throughput technologies. However, an increasing number of studies using these novel technologies are now being conducted to discover the molecular alterations behind the colorectal cancer and to find novel biomarkers and therapeutic targets [11,12,13,14]. In addition, international coordinated efforts are being made to study different tumors. In the case of colorectal cancer, The Cancer Genome Atlas (TCGA) Network has recently published a comprehensive multi-level high-throughput analysis [15]. Data from mutation rate, methylation profile, expression, and copy number variation, revealed the presence of distinctive molecular patterns. Among the tumors analyzed, 16% showed an elevated rate of mutations (the so-called hypermutated tumors), features mostly associated with MSI presence and, to a lesser extent, with somatic mutations in DNA mismatch repair or polymerase epsilon aberrations. Although many different alterations were found across the analysis, it was surprising to discover that the majority of such molecular lesions were concentrated in a few signaling pathways. The Wnt pathway is the most affected one, with alterations found in 93% of tumors regardless of subtype (hypermutated and non-hypermutated). Other commonly altered pathways in colorectal tumors were the RAS-MAPK pathway, with *KRAS*, *NRAS*, and *BRAF* genes with already known mutations, and the PI3K pathway. The hypermutated tumors were enriched in genomic alterations in genes from the TGF-beta pathway, while mutations in non-hypermutated tumors primarily affected genes from the p53 pathway. Regarding the transcriptional profile, tumors were classified into three distinct subgroups, two of which were respectively associated with MSI/CIMP and CIN features, while the third subgroup showed a mesenchymal invasive phenotype. Moreover, nearly all tumors analyzed show alterations in the expression of MYC transcriptional targets, probably as a result of increased expression and activity of MYC factor.

Special attention should be paid to the fact that two of the previously commented basic ways of alteration in colorectal tumors, the MSI and CIMP pathways, are related to epigenetic modifications, indicating the important role of the malfunction of epigenetic mechanisms in the development and clinical behavior of colorectal tumors. Epigenetic marks occur widely across the genome and extensively regulate the expression of the multitude of genes. The disposition and dosage of these epigenetic marks are strictly controlled in normal non-neoplastic cells but, in cancer cells, their deregulation opens the door to the expression or silencing of oncogenes and tumor suppressor genes, respectively. Therefore, the altered epigenome is a hallmark for many tumors, including colorectal cancer, and constitutes an important cause of heterogeneity. In fact, although the most studied and the major epigenetic events which are believed to play critical roles in colorectal cancer are CpG island methylation and histone modifications [16], there are multiple ways for epigenetic modification, including nucleosomal occupancy and remodeling, chromatin looping, and noncoding RNAs [17]. Consequently, epigenomic modifications represent an attractive target for epidemiological analysis, molecular pathology, therapeutic response evaluation, and drug design [17,18,19]. However, like the analysis of other molecular traits, the study of epigenome can be remarkably challenging due to the variability existing not only between individuals but also between and within tumors. Therefore, future development of powerful technologies to analyze the epigenomic landscape together with novel tools to integrate this information with other molecular data could help to advance the diagnosis, management, and treatment of patients.

An additional source of heterogeneity resides in the tumor microenvironment (extracellular matrix, supporting stromal cells, and immune cells) and host-tumor interactions, happening not only within tumors but also in the whole organism as a result of the spreading of cancer cells [20,21,22]. These interactions depend largely on the genetic composition of normal non-neoplastic cells and therefore, the clinical behavior of apparently similar tumors can differ among persons due to differences in genetic background and genomic variations. Moreover, the phenotypic manifestation of these genetic/genomic variations can be also modified by exposures to different insults during the individual’s lifetime. Several alterations such as stress, comorbidities, hormonal changes, inadequate lifestyle and dietary intake, as well as harmful environmental threats can contribute to the generation of otherwise not developed tumors or poor outcome of such tumors once developed. In fact, from findings in recent studies it is becoming clear that parameters such as dietary fibre intake [23], vitamin D, and blood lipid levels [24,25], adult weight [26], and sedentary habits [27] have an influence on the prevention of colorectal cancer development.

Taken together, all these factors make each tumor unique with singular characteristics from the point of view of clinical course, molecular profile, microenvironment, and host-tumor interactions: this is the so-called “unique tumor principle”. Thus, the current classification of colorectal cancer (and other tumors) in a limited number of subtypes should be abandoned and a move made towards the molecular characterization of each tumor as a single entity in order to advance in the implementation of precision oncology [28].

## 3. Novel Molecular Classifications of Colorectal Cancer

During recent years, several attempts of comprehensive molecular classifications based on transcriptomic profiles have been proposed, and a number of molecular signatures with potential clinical usefulness have been defined for the stratification of patients with different prognosis and treatment response [29,30,31,32,33,34,35,36,37,38,39]. However, to date only two molecular signatures have been approved by the FDA (Food and Drug Administration) for their clinical use in the identification of stage II/III colorectal cancer patients at risk: Coloprint™ [40] based on the expression of 18 genes, and Oncotype Dx Colon Cancer Test™ based on the analysis of expression of 12 genes [41]. Other classification proposals have not yet been implemented since (i) they lack further clinical studies supporting their usefulness; and (ii) many of such classifications are still too complex to be applied to the clinical setting due to the limited implementation of high throughput technologies in pathology laboratories. However, two recent studies [42,43] have generated promising simplified molecular signatures that can be easily tested using conventional low throughput techniques (such as immunohistochemistry and qPCR).

In one of these studies, De Sousa E Melo and co-workers [42] investigated the transcriptomic profiles (stored in different on-line repositories) from 1100 colorectal cancer patients. Using bioinformatic tools, the authors classified 90 stage II colorectal tumors in three large subtypes named CCS1, CCS2 and CCS3 (CCS, Colorectal Cancer Subtype). Interestingly, two of these subtypes were associated with previous genetic/prognostic classifications, since CCS1 tumors showed similarities with CIN/MSS tumors and CCS2 tumors were principally MSI/CIMP tumors. However, CCS3 tumors displayed important heterogeneity in their phenotypic and genetic characteristics and therefore, they did not fit in any of the previously known genetic subtypes. This led the authors to suggest that CCS3 tumors represent a novel colorectal cancer subtype, which additionally has an aggressive behavior reflecting their poor disease-free survival. Indeed, some genes whose expression is associated with CCS3 tumors are also included in the risk signature of Oncotype Dx Colon Cancer Test™. Finally, the majority of CCS3 tumors are cetuximab-resistant regardless of KRAS mutational status, as deduced from data from metastatic colorectal cancer (mCRC) patients.

In another study, Sadanandam and co-workers [43] made a combined analysis of transcriptomic data from four independent previous studies encompassing 1290 colorectal cancer patients. By bioinformatics analysis, the authors were able to identify five homogeneous colorectal cancer groups on the basis of their expression profiles. Interestingly, these expression profiles also showed important similarities with the expression patterns of different cell subtypes found in normal colonic crypts. In consequence, the colorectal cancer subtypes were named as enterocyte, goblet, inflammatory, transit-amplifying, and stem cell subtypes. This classification of colorectal tumors has important prognostic and therapeutic implications, since different subtypes display different disease-free survival and different response to chemotherapy and targeted agents (irinotecan and cetuximab).

While many of these molecular classifications could be in the future translated to clinical use after further validation, the multiple proposals of classifiers derived from different sources, data and approaches represent an additional issue. In order to circumvent these possible concerns, a colorectal cancer subtyping consortium (CRCSC) has been recently created in an attempt to construct a unified molecular classification [44]. After the analysis of 30 patient cohorts encompassing over 4000 samples, the CRCSC was able to establish four significantly homogeneous colorectal cancer molecular subtypes (CMS1-4) and a fifth subgroup without clear assignment. This was possible because different classifications generated in different works show compatible clinical, pathological and molecular characteristics. On the basis of these common features, the CMS1 tumors showing MSI/CIMP, immune infiltration, and hypermutated phenotype, may be easily related to the CCS2 class from De Sousa *et al.* [42] and to the inflammatory tumors identified by Sadanandam *et al.* [43]. Epithelial, MSS, and CIN tumors were grouped into the CMS2 consensus class, which can be associated with the CCS1 subtype from De Sousa *et al.* [42] and with the enterocyte and/or transit-amplifying subtypes from Sadanandam *et al.* [43]. Likewise, other MSS/CIN tumors classified into the CMS4 subtype share the presence of a mesenchymal phenotype with the CCS3 tumors defined by De Sousa *et al.* [42] and with the stem cell-like subgroup from Sadanandam *et al.* [43]. However, some colorectal tumors cannot be classified into the predefined subgroups and therefore, the consensus classification needs further refinement. One possible issue in the current classification may be the exclusive use of transcriptional data; perhaps, the inclusion of data from other molecular levels could contribute to the improvement of such a classification. In addition, future classifications of colorectal cancer should include the analysis of other samples beyond primary tumors, such as metastatic tissue and blood, and studies on the host-tumor interactions (that is, the influence on tumors of the immune system and the microenvironment) and cell-to-cell variability. The ideal classification should also include the analysis of inter-individual heterogeneity in the context of “unique tumor principle” taking into account both endogenous and exogenous factors involved in the peculiarity of each patient. Endogenous factors as patients’ genetic/genomic background, and exogenous factors as the exposure to environmental elements, lifestyle, and dietary intake should be incorporated into forthcoming colorectal cancer classifications.

## 4. Cell-to-Cell Heterogeneity in Colorectal Cancer

The existence of molecular similarities between normal cell types in colonic crypts and their malignant counterparts in colon cancer can be easily inferred from the analysis performed in one of the above-mentioned studies [43]. This link was already postulated in other previous study using single cells isolated from normal and tumor tissues [45]. In that work, the molecular patterns found in the different subtypes of single cells within normal crypts were similar to the molecular features of different tumors, depending on the preponderant cell subtype present in each tumor. An additional conclusion was derived from the analysis made in this work: the heterogeneity in the cell composition of colorectal tumors, at least at transcriptional level, arises from a differentiation process similar to that occurring in normal tissue. This finding is supported by the fact that the cell composition of a xenograft generated from a single cell recapitulates the cell composition found in the original tumor from which the single cell was isolated. Moreover, all these results are in agreement with the hypothesis [2] by which stem-like cancer cells can undergo a differentiation process similar to that occurring with stem cells in normal tissues.

In addition, cancer cells may transit by many different phenotypic and molecular states in the course of tumor growth [46]. Proof of this fact is the divergence found in a recent study [47] between the transcriptional profile of single cells and large cell populations (from which single cells were isolated) in breast cancers. This marked cell-to-cell heterogeneity within tumors may be an evolutionary-like mechanism by which otherwise residual cell populations could overgrow in response to an external insult, such as drug administration [48]. Therefore, cancer cell heterogeneity may play a critical role in the generation of drug resistance and ultimately in the appearance of relapses. Taken together, these findings underscore the necessity of fostering the development of analytical technologies in order to achieve the capacity to test thousand or hundreds of thousands of samples (in this case, single cancer cells) both at multiple biological levels and time points.

## 5. Immunological Heterogeneity in Colorectal Cancer

In addition to the variability in morphological, phenotypic, functional, or genetic characteristics, the heterogeneity in colorectal cancer can be also found in aspects related to the expression of molecules involved in antitumor immune-surveillance, specially the major histocompatibility complex (MHC). In colorectal cancer, from the earliest stages of carcinogenesis, clones of tumor cells exhibit altered expression of MHC antigens [49]. These clones may have different types of alterations allowing immune-selection during tumor progression [50]. This process is called immuno-editing and includes a “removal phase” of susceptible tumor cells, a second phase of “equilibrium”, and a third phase of “escape” in which clones of tumor cells “hidden” to the immune system can evade the immunological control and initiate the metastatic spreading.

Regarding MHC, the clones of tumor cells can present two types of alterations [51] that explain the variable response to immunotherapies and clonal selection during tumor progression: reversible (the so-called “soft”) and structural-irreversible (the so-called “hard”). The heterogeneity in antigen expression or genetic abnormalities in colon cancer can be seen not only in MHC but it is also present in other important molecules for immunological regulation, such as Galectin-3 or Programmed death ligand-1 [52]. Interestingly, in colon adenocarcinomas, the type and activity of peritumoral inflammatory infiltrate seems to be more related to the presence of MSI than alterations or level of expression of these molecules [53]. Clearly, the understanding of diversity and heterogeneity detected in immune molecules regulating tumor-host interactions (such as MHC) can represent one of the main challenges to overcome in the future development of immunotherapies for colon cancer.

The importance of microenvironment and host-tumor interactions, and specifically the interactions between immune cells and tumor cells, has been highlighted in previous observations in colorectal cancer with MSI phenotype in which tumor-infiltrating lymphocytes were associated with good prognosis [9]. In line with this, there is numerous evidence suggesting the preponderant role that CD3+, CD8+, and CD45RO+ cells play in antitumor responses, and the prognostic usefulness of CD8+/CD45RO+/FOXP3+ lymphocytic subpopulations. Taking into account these data, a novel prognostic index has been proposed, named Immnoscore, based on the enumeration of CD3+/CD45RO+, CD3+/CD8+, or CD8+/CD45RO+ lymphocyte populations in the invasive front and in the core of the tumor. Thus, the Immunoscore ranges from 0 (I0) to 4 (I4) depending on the density of the lymphocyte populations found in both regions of lesion. This score shows strong association with several prognostic measurements, including disease-free survival, disease-specific survival, and overall survival: the higher the Immunoscore, the better the prognosis (reviewed in [54]). The Immunoscore has been demonstrated to be effective even in the determination of the risk of relapse in early-stage (TNM stages I/II) colorectal cancers [55]. Indeed, in CRC tumors with TNM stages I/II/III, the immune pattern was superior to the classical TNM classification in the prognostic prediction [56]. Therefore, in order to better determine the prognosis of cancer patients, to better identify patients at risk of relapse, and to better allocate patients to receive adjuvant therapy, the incorporation of the Immunoscore to the current TNM staging system to generate the TNM-I [54,57] has been suggested.

## 6. Heterogeneity in Disseminated and Metastatic Disease

In colorectal tumors, the heterogeneity is not only limited to primary lesions but also metastases and circulating and disseminated cancer cells may harbor different levels of variability. During the tumor growth, cancer cells detached from primary tumors reach the bloodstream and become circulating tumors cells (CTCs). Before their intravasation into blood vessels, cancer cells must undergo the de-differentiation process known as epithelial-to-mesenchymal transition (EMT) in order to attain the mesenchymal phenotype necessary for their migration and infiltration in surrounding mesenchymal tissues [58]. Once in the blood, the phenotype of CTCs is highly variable and they can assume roles typical of mesenchymal cells, epithelial cells or both [59]. CTCs with mesenchymal phenotype are believed to have enhanced capacity to form metastasis through extravasation and generation of secondary tumor deposits [60]. Therefore, the analysis of heterogeneity in CTCs could help to predict metastasis occurrence, disease progression or response to therapy [61], and perhaps in the future, even could help to prevent distant tumor dissemination.

In line with this, current research is moving on the right track exploring the usage of novel sample sources, including among others, plasma and serum [62]. As main advantages, these samples can be obtained through low-invasive procedures and can be used to perform analysis at multiple time points in order to monitor disease evolution and/or treatment response. This fact is illustrated by the detection of *KRAS* mutations in circulating free DNA (cfDNA) in serum of mCRC patients who developed panitumumab resistance [63,64,65]. Moreover, *KRAS* mutations were detected in 59% of tumor samples from mCRC patients refractory to chemotherapy and targeted agents (bevacizumab and anti-EGFR), whereas the same mutations were detected in 69% of cfDNA samples. Higher frequency of *BRAF* and *PIK3CA* mutations in cfDNA were also found regarding matched tumor samples. The increased frequency of mutations in cfDNA might reflect the emergence of mutant subclones during treatment, under-represented when the primary tumor was extracted and evaluated. How this intra-tumor heterogeneity in low-copy mutant subclones affects the clinical response to panitumumab and cetuximab remains controversial [66,67,68].

In addition to DNA, plasma and serum also contain relevant information in other molecular specimens, such as microRNAs [69] and proteins [70], and therefore, they can be interesting options to study cancer metabolism and secreted non-coding RNAs and proteins as well as to discover novel cancer biomarkers [71]. However, most of the works performed with high throughput technologies in cell line models, tumor tissues, and alternative sample sources, have been focused on the analysis of whole genome and transcriptome, while few have had studies addressing the investigation at other molecular levels, such as proteins, metabolism or secretome [72,73].

Since the cell composition and molecular characteristics of metastatic lesions might significantly differ from those found in primary tumors, the analysis of heterogeneity in metastasis could provide relevant information regarding outcome, response to treatment and management of patients with advances tumors. This fact has special relevance for the discovery of prognostic and predictive biomarkers, which are usually developed and tested in primary tumors. Given that metastases are generated by cells or clones that, although under-represented in the primary tumor, display selective advantage to grow at secondary sites [74,75]. The usefulness of biomarkers designed in primary tumors may be potentially compromised in the case of metastases. In fact, even in the case of existing similarities between the primary tumors and metastases, the latter can develop and accumulate alterations considerably different to those present in primary tumors [2]. However, the predictive molecular markers so far implemented for the stratification of mCRC patients susceptible to respond to anti-EGFR antibodies (*KRAS* and *NRAS* mutations) have been demonstrated as being highly concordant between metastases and primary tumors [76,77,78]. Furthermore, at least in the case of colorectal tumors, this concordance is mostly maintained when recurrent colorectal cancer-specific mutations (from which potential novel biomarkers may be developed) are analyzed by targeted and whole genome next-generation sequencing [79,80,81,82]. In addition to the genomic heterogeneity, within liver metastases of colorectal tumors it is also possible to distinguish variable patterns at the proteomic level. While the external region of metastases is enriched in proteins involved in proliferation, migration, and drug metabolism, the core of lesions shows increased carbohydrate metabolism and DNA-repair activity [83], in agreement with the physiologically specialized organization proposed for tumors [84].

## 7. Clinical Implications of Heterogeneity in Metastatic Disease

Both inter-tumor heterogeneity (colorectal cancer subtypes) and intra-tumor heterogeneity (cellular heterogeneity within individual tumors) may have implications on the prognosis, choice of treatment, and emergence of resistances. Oncogenic mutations of components of the RAS-RAF-mitogen-activated protein kinase (MAPK) pathway are common in colorectal cancer. Different clinical trials have indicated that a positive effect of anti-EGFR blockage is restricted to patients with *RAS* wild type tumors [85]. *KRAS* mutations at exon 2 (codons 12 and 13) are found in 45%–50% of mCRC. In addition, a subgroup of 14.7%–17.4% of patients with *KRAS* exon 2 wild-type mCRC may harbor significant mutations at other *KRAS* exons (exon 3 at codon 61, and exon 4 at codons 117 and 146) and/or in the *RAS* isoform *NRAS* at the same codons assessed in *KRAS*. Mutations on *BRAF* have been found in 8%–15% of colorectal cancer and they are mutually exclusive with KRAS mutations. *BRAF* mutation is clearly associated with a worse prognosis and, at least in pre-treated patients, with a poor response to anti-EGFR therapy [86,87]. *KRAS* status has been also defined as a prognostic factor in mCRC patients treated in the first line with oxaliplatin-based chemotherapy and bevacizumab [88]. In these patients, the presence of mutations at *KRAS* exon 2 was independently associated both with poor progression-free and overall survival.

The addition of panitumumab to oxaliplatin-based chemotherapy in first line treatment of mCRC was explored in the PRIME trial. Among patients without *RAS* mutations, the combined treatment with panitumumab and FOLFOX significantly improved both the progression-free survival and overall survival. In patients receiving FOLFOX4 plus panitumumab, any *RAS* mutations were negatively associated with outcomes [89]. Also, the reassessment of data from the CRYSTAL trial demonstrated that the addition of cetuximab to irinotecan-based chemotherapy in the first-line treatment of mCRC patients improves response rate, progression-free survival, and overall survival in the *RAS* wild-type subset [90].

Interestingly, some patients harbor tumors with low-prevalence mutations (between 0.1% and 5% of mutant DNA to wild type). It has been suggested that selective growth of these mutated subclones could be related to clinical tumor progression. Mathematical modeling and clinical data from plasma cfDNA of mCRC patients demonstrated that most radiographically apparent lesions may contain at least 10 resistant subclones [91]. Expansion of resistant subclones could be associated with lack of response to anti-EGFR in the clinical setting. These facts were described for an acquired *EGFR* ectodomain mutation [92] and for the appearance of new *KRAS* mutations [63,64].

A number of novel targeted drugs are now in clinical trials in mCRC patients [93]. Clearly, selection of patients based on intrinsic subtypes of colorectal cancer but also taking into account intra-tumor heterogeneity offers the best chance to find an optimal therapy (Table 1). Thus, the discovery of new potential active targeted agents is currently driven by identification of actionable mutations in mCRC. Different genomically driven clinical trials and profiling studies in mCRC and other tumors are in progress or have been recently completed [5,94]. A huge number of different drugs specifically designed against several receptors and signal transduction pathways, including tyrosine kinase inhibitors and monoclonal antibodies against receptors from ERBB family (EGFR, HER2 and HER3), PI3K/AKT/mTOR inhibitors, MET/HGF targeted drugs, and MAPK pathway inhibitors (such as BRAF inhibitors), are currently in diverse phases of clinical development.

**Table 1:** Sources of genetic heterogeneity known to predict outcome/response to drugs currently administered to colorectal cancer patients.

Genetic Source	Heterogeneity	Drug	Clinical Significance	Sample Source	Analysis	References
RAS (KRAS, NRAS)	Mutations	Anti-EGFR antibodies	Predictive	Primary and metastatic tissue, CTC, cfDNA	Next-generation and Sanger sequencing, BEAMing^®^, high-performance liquid chromatography, dropled dPCR, qPCR	[61,62,63,78,79,89,95,96,97,98,99]
BRAF	Mutations	Chemotherapy and targeted agents	Prognostic, possible predictive (anti-EGFR antibodies)	Primary and metastatic tissue, cfDNA	Next-generation and Sanger sequencing, high-performance liquid chromatography, BEAMing^®^, qPCR	[62,78,89,96,97,100,101,102,103]
MMR system (e.g., MLH1 gene)	Mutations (hereditary CRC) or CpG island methylation (sporadic CRC)	Chemotherapy in adjuvant setting	Prognostic, possible predictive to adjuvant 5-FU-based regimens	Primary tissue	IHC, (q)PCR	[9,103,104,105,106,107,108,109,110,111]
PI3K	Mutations	Anti-EGFR antibodies	Possible predictive	Primary and metastatic tissue, cfDNA	Next-generation and Sanger sequencing, BEAMing^®^, qPCR	[78,96,112]
cMET	Expression	Anti-EGFR antibodies	Possible prognostic and predictive	Primary and metastatic tissue	Expression microarrays, IHC	[43,113,114]
EGFR	Mutations, amplifications	Anti-EGFR antibodies	Possible predictive	Primary and metastatic tissue, cfDNA	Next-generation and Sanger sequencing, BEAMing^®^, qPCR, FISH	[62,92,115]

## 8. Future Directions

The biological interpretation of the large amount of data obtained from the previous analyses represents an important challenge to overcome in the coming years. In order to achieve full understanding of the pathological processes underlying the malignant transformation of cancer cells as well as to develop tailored therapies and prognostic and predictive biomarkers for the precision medicine era, we need to go further in the integration of molecular data generated by high throughput technologies. This integration could allow the finding of cause-effect links between the different molecular levels analyzed by “omics”. The discipline that pursues the previous objective is systems biology and its application to the study of tumors is cancer systems biology [116,117].

However, despite some slight advances [118,119], cancer systems biology has still far to go to provide the necessary solutions for the previous issues. In the near future, with the development of new throughput levels in analytical technologies and the enhancement of cancer systems biology approaches, we could go toward the full implementation of precision medicine in oncology. An additional step is necessary to fulfill this objective: the information generated by cancer systems biology should be transferred to patients, first through its application in properly designed clinical trials, and later in tailored clinical decision-making (Table 2).

**Table 2:** Challenges to overcome in the analysis of cancer heterogeneity and classification.

Challenge	Possible Solution
Inter-patient variability	Achieve full knowledge and understanding about the variability between individuals and how this variability contributes to disease
Inter-tumor variability	Classification of the diverse types of tumors from the point of view of common phenotypic, clinical and molecular features
Intra-tumor heterogeneity	Novel analytical techniques and devices must be developed in order to increase the resolution of current high-throughput technologies and make possible the entire analysis of all cells within tumors
Design of precise/personalized anticancer drugs	Anticancer therapies must be designed based on deep analysis of tumors and their intrinsic heterogeneity
Novel design of clinical trials	Clinical trials must include multi-level high-throughput analysis to define the responsiveness of different patients, tumors, and even cells within tumors
Technological barrier	Design of affordable and simple technologies to make possible their clinical implementation
Analysis and integration of data	Development of cancer systems biology in order to generate models to obtain understandable and useful data for clinicians and patients
